# Quality of life outcome study of children that had undergone surgery for oesophageal atresia with or without a tracheo-oesophageal fistula

**DOI:** 10.1007/s00431-026-06906-6

**Published:** 2026-04-14

**Authors:** Niamh M. Edwards, Prabhu Sekaran, Semiu E. Folaranmi

**Affiliations:** 1 School of Medicine, Cardiff University, Cardiff, UK; 2 Department of Paediatric Surgery, Noah’s Ark Children’s Hospital for Wales, Cardiff, UK

**Keywords:** Oesophageal atresia, Surgery, Quality of life

## Abstract

Oesophageal atresia/tracheo-oesophageal fistula (OA/TOF) can lead to early complications such as premature delivery, feeding problems, and mortality, meaning immediate postnatal surgery is required. This retrospective study aims to evaluate the quality of life (QoL) of patients operated for OA/TOF in South Wales over the last 10 years using a modified version of the Gastrointestinal Quality of Life Index (GIQLI) to assess QoL. The GIQLI explores different aspects of QoL, including symptoms, physical, emotional, and social wellbeing. Participants’ parents were contacted over the phone inviting them to participate in this study, with the 35 questions asked over the phone or sent to participants’ parents by email. Each question has an option of 5 answers scored 0–4, with a higher score given to the answer indicating better QoL. The maximum possible score for this study with 35 questions was 140. Thirty-eight patients took part in this study, with an overall mean GIQLI score of 106 out of 140 (76%).

*Conclusion:* The findings indicate that OA/TOF patients have adequate post-surgical quality of life. However, this study was limited by a lack of previous research, as well as a limited number of participants for types A, B, D, and E.

**Table:** 

**What Is Known:** *• Oesophageal atresia/tracheo-oesophageal fistula (OA/TOF) is a condition requiring immediate postnatal surgery and is associated with significant complications in early life.* **What Is New:** *• This data collected in this study indicates that OA/TOF patients who have undergone surgery in South Wales have an adequate quality of life.* *• This study adds new data into a limited area of research.*

**Supplementary Information:**

The online version contains supplementary material available at 10.1007/s00431-026-06906-6.

## Introduction

Oesophageal atresia (OA) is a congenital abnormality characterised by discontinuation of the oesophagus, with or without a tracheo-oesophageal fistula (TOF) [1]. There are 5 subtypes of OA (types A–E), with types B–E involving a TOF [2]. Eighty-nine percent of cases of OA in Wales have an associated TOF [3].

In Wales, OA/TOF is the most common congenital gastrointestinal abnormality—occurring in 3.2 of every 10,000 births per year [3].

OA/TOF occurs from the fourth week of pregnancy as a result of incomplete embryonic compartmentalisation of the foregut into its gastrointestinal and respiratory segments [4]. It is diagnosed antenatally (in 42% of Welsh cases) and postnatally (in 58% of Welsh cases) [3]. Multiple features point towards the diagnosis of OA/TOF, for example, antenatally, through the presence of polyhydramnios on ultrasound in the 3rd trimester and postnatally, through the inability to pass a nasogastric tube into the stomach [5].

OA/TOF can lead to a wide range of early complications such as premature delivery, feeding problems, poor weight gain and even death in some cases—especially where other associated congenital abnormalities are present [5]. Surgery is therefore required soon after birth to correct the anomaly and prevent long-term complications, ensuring that the infant can self-ventilate, feed, and thrive in the long term. Post-surgery complications can occur such as gastro-oesophageal reflux, oesophageal anastomotic stricture, recurrent chest infections, and tracheomalacia [5], but the overall prognosis after surgery is good, with a survival rate of 90% in those without major cardiac anomaly [4]. Most children born with OA/TOF can then go on to live relatively normal lives [4].

This study aims to evaluate the quality of life (QoL) of patients born with OA/TOF in South Wales post-surgery.

## Ethics approval and consent to participate

The study proposal was submitted to the Research Ethics Board of Cardiff and Vale University Health Board prior to commencement. The committee reviewed the submission and determined that the study did not require formal ethical approval. All procedures performed in this study were conducted in accordance with the ethical standards of the institution and are also in accordance with the 1964 Declaration of Helsinki and its later amendments. Informed consent to participate was obtained verbally over the phone from all participants’ parents prior to their inclusion in the study.

## Methods

QoL was assessed using a modified version of the Gastrointestinal Quality of Life Index (GIQLI), a well-recognised questionnaire used to assess QoL specifically in patients with gastrointestinal disease first developed by Eypasch et al. in 1995 [6]. It consists of 36 questions overall with 5 domains covered within these questions—core and disease-specific symptoms (which in this study were combined into one category—GI symptoms), as well as physical, emotional, and social wellbeing [6].

Thirty-five out of 36 questions of the GIQLI were included in this study, with the question ‘To what extent has your sexual life been impaired (harmed) because of your illness?’ excluded due to the age group of our patient population. Whilst the GIQLI was developed for adults, it has been used in other paediatric studies due to its wide range of questions and its ability to explore the impact of disease-specific symptoms.

Each question in the modified GIQLI had an option of 5 different answers (with 26 of the 35 questions having possible answers of All of the time, Most of the time, Some of the time, A little of the time, Never) and are scored 0–4 with a higher score given to the answer indicating a better QoL. The total score of all of the questions is then added up; with a higher overall score indicating a better QoL [6]. The maximum possible score for this study with 35 questions was 140—with the individual domains having a maximum possible score of 76 for GI symptoms, 20 for emotional wellbeing, 28 for physical wellbeing, and 16 for social wellbeing.

Over a period of 4 weeks, parents of children who had had surgery for OA/TOF over the past 10 years in all South Wales health boards were contacted directly over the phone at least twice, inviting them to participate in this study. Informed consent was obtained from legal guardians, and after this, the GIQLI questionnaire was either asked over the phone or sent to them by email, using the GIQLIs questions as the surveys guideline.

Whilst answering the questionnaire, some parents felt that some of the questions were not relevant to their child. For example, the question ‘How often during the past 2 weeks has your child had trouble swallowing their food?’ was not relevant to children who were fed via a nasogastric tube. In cases like this, their questionnaire score was adjusted by being divided by the number of questions answered in total (excluding the ones they did not think were relevant to their child) and were then multiplied by 35 (the total number of questions in the questionnaire). This then meant that despite the fact that they did not answer all of the questions, their overall score was still comparable to scores where all 35 questions had been answered.

Statistical analysis was performed using an unpaired *t*-test to determine if there were significant differences between the different groups in terms of their QoL scores, with a *p* value < 0.05 deemed to be significant.

## Results

Fifty-six patients were identified who were eligible to take part in the study, with 38 out of 56 questionnaire responses collected in total (68%). Of those who did not respond—8 consented to taking part in the study but did not respond to the questionnaire when emailed, 4 patients could not be reached over the phone after 3 phone calls, 3 patients were excluded, and 3 declined taking part in the study. Thirty of the participants underwent primary repair of their OA/TOF, 3 underwent delayed repair, 3 underwent staged repair, and for 3 participants, their method of repair is unknown. Of those who underwent a staged repair 2 of these underwent a gastric pull-up and 1 participant’s staged repair method is unknown. Of those who underwent primary repair, 13 underwent further oesophageal dilatation due to a subsequent development of an oesophageal stricture.

Of the 38 participants in the study, 32 had type C OA/TOF, 2 individuals each had types A and D, and 1 individual each had types B and E.

The ages of the participants were split into 5 categories—0–2 years, 3–4 years, 5–6 years, 7–8 years, and 9–10 years. The age category with the largest number of participants was 5–6 years, with 10 participants in this group, followed by 8 individuals in the 7–8 years age group, 7 individuals each in both the 9–10 years and 3–4 years age groups, and 6 individuals in the 0–2 years age group. The mean age of the participants was 5.5 years old.

Twenty-one of the participants were assigned Female at birth, whilst the other 17 were assigned Male at birth. The full demographics of all participants can be seen in Table 1.

**Table 1:** Participant demographics

Participant number	Gestational age (weeks)	Sex (M/F)	Birth Weight (g)	Spitz category	Type of atresia	Type of repair	Length of NICU stay (days)	Polyhydramnios (yes/no)	Prenatal diagnosis (yes/no)
1	38 + 0	F	2770	1	C	Primary	9	Yes	No
2	31 + 6	F	1650	1	C	Primary	Unknown	Yes	Yes
3	38 + 0	F	2730	Unknown	C	Primary	Unknown	Yes	No
4	Unknown	M	Unknown	Unknown	C	Primary	Unknown	Unknown	Unknown
5	37 + 0	F	3320	1	B	Staged (gastric pull-up)	77	Yes	Yes
6	42 + 0	M	3490	1	C	Primary	4	No	No
7	40 + 0	F	3380	1	C	Primary	3	No	No
8	37 + 5	F	2200	2	C	Primary	7	Yes	No
9	39 + 4	F	2370	1	C	Primary	9	Yes	Yes
10	28 + 0	M	2000	Unknown	C	Primary	Unknown	Unknown	Unknown
11	28 + 5	M	1210	2	A	Staged (gastric pull-up)	208	Yes	Yes
12	41 + 3	M	3180	1	C	Primary	5	Yes	No
13	39 + 2	M	3900	1	C	Primary	16	No	No
14	39 + 3	F	2680	1	C	Primary	5	No	No
15	36 + 5	F	2490	2	C	Primary	Unknown	No	No
16	31 + 2	M	1830	1	C	Primary	Unknown	Yes	No
17	30 + 4	F	1300	2	C	Unknown	Unknown	Unknown	No
18	40 + 4	F	3140	1	C	Primary	4	Yes	No
19	36 + 6	F	2200	1	C	Primary	Unknown	No	Unknown
20	32 + 4	M	1750	1	C	Primary	10	Yes	Yes
21	36 + 0	F	2370	1	A	Delayed Primary	156	Yes	Yes
22	39 + 6	M	3240	1	C	Delayed Primary	13	Yes	No
23	34 + 3	F	1925	1	C	Delayed Primary	198	Yes	No
24	39 + 0	M	3850	1	D	Primary	20	No	No
25	37 + 3	F	2620	1	C	Primary	51	Yes	Yes
26	31 + 3	M	1700	2	C	Primary	31	No	No
27	34 + 5	F	1600	1	C	Primary	85	No	No
28	31 + 0	F	1320	2	C	Primary	12	No	No
29	39 + 1	F	3130	1	D	Primary	10	Yes	No
30	41 + 0	F	3390	1	C	Primary	6	No	No
31	38 + 1	M	3010	1	C	Primary	5	No	No
32	39 + 6	M	2600	1	C	Primary	15	No	Yes
33	37 + 0	F	2400	1	C	Primary	27	Yes	No
34	34 + 5	F	1972	1	C	Primary	15	No	No
35	39 + 0	M	3880	1	C	Primary	4	No	No
36	38 + 5	M	Unknown	Unknown	C	Unknown	Unknown	Yes	Unknown
37	39 + 0	M	Unknown	1	E	Staged (unknown method)	Unknown	No	No
38	38 + 2	M	3360	1	C	Primary	13	No	No
Mean	36 + 5	/	2570	/	/	/	36.4	/	/
Standard deviation	± 3 + 5	/	± 776	/	/	/	± 57.5	/	/
Binary outcomes	/	M—17 (44.7%) F—21 (55.3%)	/	1—28 (73.7%) 2—6 (15.8%) Unknown—4 (10.5%)	A—2 (5.3%) B—1 (2.6%) C—32 (84.2%) D—2 (5.3%) E—1 (2.6%)	Primary—30 (78.9%) Delayed primary—3 (7.9%) Staged—3 (7.9%) Unknown—2 (5.3%)	/	Yes—18 (47.4%) No—18 (44.7%) Unknown—3 (7.9%)	Yes—8 (21.1%) No—26 (68.4%) Unknown—4 (10.5%)

The overall mean GIQLI score of the 38 participants was 106 out of a maximum of 140 (76%). With the mean scores of the 4 categories of the questionnaire ranging from 76 to 81% as seen in Table 2.

**Table 2:** Mean scores of the 4 different categories of the GIQLI questionnaire

GIQLI domain	Mean score		Percentage
GI symptoms	58	out of 76	76%
Emotional	16	out of 20	80%
Physical	20	out of 28	71%
Social	13	out of 16	81%

**Fig. 1:**
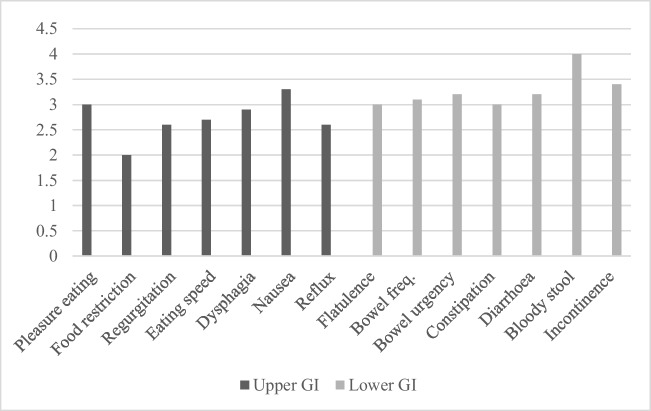
Summary of mean scores of upper GI and lower GI symptoms categories of the modified GIQLI

The overall mean score of the GI symptoms domain was 58 out of 76 (76%). The GI symptoms category was further separated into an upper GI symptoms category (Fig. 1a) and lower GI symptoms category (Fig. 1b). The upper GI symptom category had a mean score of 2.7 out of 4, with the symptom with the worst overall score being food restriction—the score being 2.1 out of 4. The lower GI symptoms category had a higher overall mean score than the upper GI symptoms category—with a mean score of 3.2 out of 4.

**Fig. 2:**
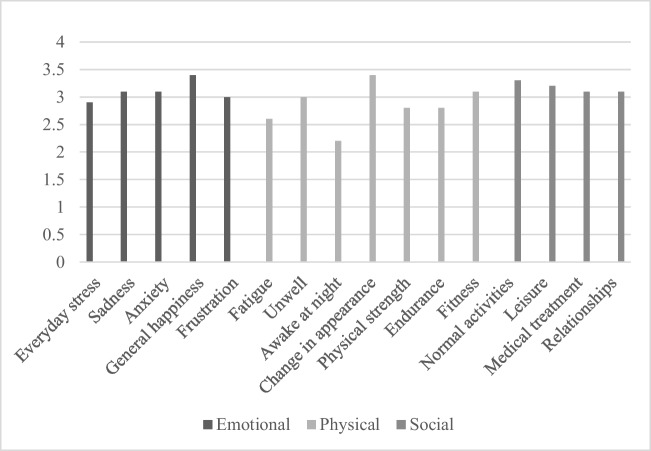
Mean scores of the emotional, physical and social wellbeing categories of the modified GIQLI questionnaire

The average score for the emotional wellbeing category was 16 out of 20 (80%). Within this category, there were 5 domains—everyday stress, sadness, anxiety, general happiness, and frustration. With the mean scores for each of these domains seen in Fig. 2a, the mean score of these 5 domains was 3.1, with the general happiness domain having the highest mean of this category with a score of 3.4 out of 4. The everyday stress domain had the lowest mean score of the 5 domains with a score of 2.9 out of 4.

The physical wellbeing category was split into 7 domains which can be seen in Fig. 2b.The overall mean score for this category was 2.8 out of 4. The domain with the highest overall mean score within this category was change in appearance, with a score of 3.4 out of 4, whilst the domain with the lowest score was waking at night with a score of 2.2 out of 4.

The social wellbeing category was the category with the highest overall mean score—with an overall mean score of 3.2 across 4 domains. The details of the 4 domains are shown in Fig. 2c.

The mean scores for the age groups of the participants ranged from 91 out of 140 (65%) to 126 out of 40 (90%). The 0–2 years age group had the lowest overall mean score whilst the 3–4 age group had the highest (Table 3).

**Table 3:** Mean scores the modified GIQLI questionnaire based on the age group of the participants

Age range	Amount	Mean score	Percentage
0–2 years	6	91	65%
3–4 years	7	126	90%
5–6 years	10	97	69%
7–8 years	8	108	77%
9–10 years	7	111	79%

With regard to gender of the participants, the overall mean score for Females was slightly higher than the Males, but did not reach statistical significance, 110 out of 140 (79%) compared to 102 out of 140 (73%), *p* = 0.13 (Supplementary Table 1).

With respect to the different types of OA, the GIQLI scores ranged from 80 out of 140 (57%) to 121 out of 140 (86%), with type D and type E having the lowest and highest questionnaire scores respectively, as demonstrated in Supplementary Table 2. The scores of patients born with type C OA/TOF (the category with most participants) were statistically analysed against types A and D, with the *p* values equating to 0.34 and 0.07 respectively. The scores of patients born with types A and D were also analysed against each other, with *p* = 0.13. OA/TOF types B and E could not be statistically analysed as they each had only one value each within them.

## Discussion

 In light of the overall mean score of the questionnaire being 106 out of 140 (76%), we can infer that the participants of the study generally have an acceptable level of QoL.

It is suggested from the scores that the most troubling GI symptom amongst OA/TOF patients is food restriction, with other symptoms such as reflux, regurgitation, slow speed of eating and nausea/vomiting also potentially contributing to general feeding issues overall. These scores were to be expected in this study—due to how common feeding issues can be in OA/TOF patients post-surgery. This result is also consistent with other studies done on OA/TOF patients, where a study by Schier et al. showed that 68% of children in an OA/TOF support group experienced some form of feeding difficulty [7].

Upper GI symptoms are suggested to affect OA/TOF patients more than lower GI symptoms overall, which was consistent with our expectations before the study due to OA/TOF being primarily an upper GI pathology and the potential post-op complications of OA/TOF repair such as recurrence of TOF, oesophageal stricture and GORD all affecting the upper GI tract [8].

The data suggests lower GI symptoms do sometimes trouble this patient group even if the effect is less potentially prevalent. This aligns with previous observations given the fact that anorectal malformations are reported in 8–11% cases of children born with OA/TOF [9] and 4 of our participants having this condition.

The emotional wellbeing of the participants is suggested to be generally good and stable, with the scores of this category suggesting that negative emotions such as anxiety and sadness affect participants sparingly.

A significant factor in the emotional wellbeing category is the general happiness domain having a mean score of 3.4 out of 4, as it integrates several different factors of a patient’s life into one domain—for example, a family life, effect of GI symptoms on day-to-day life, and quality of medical care received.

Looking at the physical wellbeing category, the scores suggest the participants do have good physical strength, endurance, and fitness. This is a positive outcome, as it demonstrates that participants are able to keep up with their peers and live a relatively normal life in this aspect. The scores also suggest that children are minimally impacted by change in their appearance, implying that they can fit into society without feeling ostracised.

The lowest score in the physical wellbeing category was the awake at night domain—suggesting that children born with OA/TOF wake up a moderate number of nights each week. However, this could also be down to sleep problems being fairly common in young children, where it was suggested in a study by Goodlin-Jones BL et al. that sleep problems affect roughly 20% of infants and toddlers overall [10].

Another domain in the physical wellbeing category with a relatively low score was fatigue—with the score suggesting it bothers participants some of the time. This could be contributed to by the unwell domain—where it was suggested that the participants also feel unwell some of the time, possibly contributing to their overall fatigue. Fatigue in children with chronic illnesses is also relatively common, as discussed in a study of 434 children with chronic illness in the Netherlands where it was found that 21.8% of participants were severely fatigued [11]. Considering this, the finding of moderate fatigue in the participants of this study could therefore be coincidental.

The results of this study suggest that the social wellbeing of the participants of the study was the best out of all of the other categories. This result suggests that the participants are able to complete normal activities (such as school or nursery) as well as their activities of leisure with the effects of their condition minimally affecting them.

The results also suggest that participants were only minimally disturbed by the medical treatment of their illness, a huge positive considering the negative effects that medical trauma can have on children. This also implies that the medical care that they received was of a high quality tailored to the overall wellbeing of the child, both physically and mentally. It can also be inferred from the social wellbeing category that being born with OA/TOF had little impact on personal relationships with friends and family.

Looking at the statistical analysis of Female and Male participants (*p* = 0.13), we can infer that there is no statistically significant difference between the quality of life of Males and Females born with OA/TOF.

This is the same case for the statistical analyses between OA/TOF types C and A (*p* = 0.34), C and D (*p* = 0.07), and A and D (*p* = 0.13).

The OA/TOF type with the lowest overall questionnaire score was type D. This could be due to the complexity of the condition with there being both a proximal and distal TOF [2], which can sometimes lead to misdiagnosis as OA/TOF type C as well as the need for multiple surgeries to repair both the proximal and distal fistulas meaning an increased risk of post operative complications overall [8]. On the other hand, the OA/TOF type with the highest questionnaire score was type E—which could be explained due to this condition having no atresia and only an isolated TOF [2] and so a potentially smaller likelihood of surgical complications.

## Conclusion

The data collected from this study suggest an acceptable level of quality of life in each category asked in the GIQLI questionnaire.

## Limitations

This study was limited by the lack of comparable data on OA/TOF patients in South Wales, as well as the small sample size of this study. There were also limitations in comparing questionnaire scores of each type of OA/TOF as there were only 1–2 individuals who answered the questionnaire who had OA/TOF types A, B, D, and E, and 32 participants in the OA/TOF type C category. This could mean that the QoL score for the OA/TOF type C group is possibly more representative and accurate overall, prompting the need for further research into the rarer kinds of OA/TOF in order to obtain more reliable and comprehensive data.

This was further highlighted when speaking to parents of OA/TOF patients over the phone and inviting them to partake in the study, where many were very thankful and excited that this research was being done—due to the overall lack of research into OA/TOF patients in South Wales.

Another limitation of this study was the reporting of symptoms, specifically in differentiating between symptoms such as reflux and regurgitation and nausea and vomiting. This is particularly relevant in the 0–2 years age category, where the children are unable to self-report these symptoms to their parents. Parents of children in this study were asked to give an answer based on direct observation of their child and possible physical indicators of these symptoms, such as fussiness and drooling associated with nausea and vomiting and hiccups and crying associated with reflux and regurgitation. However, we recognise that these observations may not be entirely accurate and may have affected the scores of the questionnaire, especially in the 0–2 years age category where scores may have been different if the child had been older and able to self-report these symptoms.

Other associated gastrointestinal anomalies such as anorectal malformation (present in 4 participants) were also a limitation to this study. Similarly to the 0–2 age category making it hard to differentiate between symptoms, anorectal malformation may have also caused this effect in differentiating between symptoms such as diarrhoea and incontinence. Parents were again asked to give an answer based on direct observation of their child and possible physical indicators of these symptoms. Or alternatively, if a parent felt that one of the symptoms were not applicable to their child (such as diarrhoea in a faecal incontinent child), their questionnaire score was adjusted by being divided by the number of questions answered in total (excluding the ones they did not think were relevant to their child) and were then multiplied by 35 (the total number of questions in the questionnaire). This then meant that despite the fact that they did not answer all of the questions, their overall score was still comparable to scores where all 35 questions had been answered.

We also recognise that intercurrent illness was a limitation to this study. A few participants had been in hospital and/or were unwell whilst this study was going ahead, meaning that their GIQLI scores were comparably lower than those of well children due to the presence of extra symptoms at the time of being asked the questionnaire.

## Supplementary Information

Below is the link to the electronic supplementary material.

ESM 1 Supplementary Material 1 (DOCX 20.2 KB)

## Data Availability

No datasets were generated or analysed during the current study.
