# Supplementary material for: Quality of life outcome study of children that had undergone surgery for oesophageal atresia with or without a tracheo-oesophageal fistula
Source: Eur J Pediatr. 2026 Apr 14;185(5):264. doi: 10.1007/s00431-026-06906-6 (PMC13079472; doi:10.1007/s00431-026-06906-6)
Supplement: Supplementary file 1 — Supplementary Material 1 (DOCX 20.2 KB) [file 431_2026_6906_MOESM1_ESM.docx]

| Gender | Amount | Mean score | Percentage |
| --- | --- | --- | --- |
| F | 21 | 110 | 79% |
| M | 17 | 102 | 73% |

Supplementary Table 1 - mean scores the modified GIQLI questionnaire based on gender of the participants

| OA/TOF type | Amount | Mean score | Percentage |
| --- | --- | --- | --- |
| A | 2 | 114 | 81% |
| B | 1 | 121 | 86% |
| C | 32 | 107 | 76% |
| D | 2 | 80 | 57% |
| E | 1 | 125 | 89% |

Supplementary Table 2 - mean scores the modified GIQLI questionnaire based on the OA/TOF type
